# Craniomandibular Trauma and Tooth Loss in Northern Dogs and Wolves: Implications for the Archaeological Study of Dog Husbandry and Domestication

**DOI:** 10.1371/journal.pone.0099746

**Published:** 2014-06-18

**Authors:** Robert J. Losey, Erin Jessup, Tatiana Nomokonova, Mikhail Sablin

**Affiliations:** 1 Department of Anthropology, University of Alberta, Edmonton, Alberta, Canada; 2 Zoological Institute, Russian Academy of Science, Saint-Petersburg, Russia; University of Oxford, United Kingdom

## Abstract

Archaeological dog remains from many areas clearly show that these animals suffered tooth fractures, tooth loss, trauma, and dental defects during their lives. Relatively little research has explored the meanings of these patterns, particularly for ancient dog remains from small-scale societies of the North. One limiting issue is the lack of comparative data on dental health and experiences of trauma among northern wolves and dogs. This paper examines tooth loss, tooth fracture, enamel hypoplasia, and cranial trauma in a large sample of historic dog and wolf remains from North America and Northern Russia. The data indicate that the dogs more commonly experienced tooth loss and tooth fracture than the wolves, despite reportedly being fed mostly soft foods such as blubber and fish. The higher rates observed in the dogs likely is a result of food stress and self-provisioning through scavenging. The ability to self-provision was likely important for the long-term history of dog use in the north. Dogs also more commonly experienced cranial fractures than wolves, particularly depression fractures on their frontal bones, which were likely the result of blows from humans. Hypoplastic lesions are rare in both wolves and dogs, and probably result from multiple causes, including food stress, disease, and trauma.

## Introduction

Remains of dogs from across the globe show that these animals experienced traumatic injuries, tooth loss, and disease during their lifetimes. Signs on the skeleton marking these experiences have been shown to have significant interpretive potential for understanding the life histories of dogs, including their relationships with people [1]–[8]. Research on skeletal pathology is still rare for archaeological dog remains from small-scale societies, particularly those of the North, despite the fact that dogs historically were common across this region's diverse array of hunting and herding cultures. One of the clear limiting factors in interpreting signs of trauma and pathology on the skeletons of northern dogs is a lack of comparative skeletal or documentary data. For example, it is often difficult to ascertain if rates of fracture, tooth loss, or dental defects in archaeological dog remains differ from those observed in local wild canids. Biologists and paleontologists have generated some useful comparative canid trauma and disease data [9]–[19], but such studies often focus on single highly specific patterns such as tooth fracture, limiting the breadth of their usefulness for archaeological interpretation. Further, most ethnographic accounts of northern groups provide relatively few details about the actual lives of the dogs in these societies, with some notable exceptions [20]–[22]. To move the study of ancient dog life histories forward, more detailed comparative studies are needed.

Archaeologists working with Late Holocene dog remains from Arctic North America have commented on the occurrence of depression fractures on crania, suggesting they represented “animals sometimes having been severely disciplined as part of their management” [23]; [24]–[25]. However, alternative causes of these types of fractures, including kicks from prey animals, should be considered, particularly if similar lesions occur at comparable rates in wolves from the same region. Other lesions on Arctic archaeological dog specimens, typically punctures in the bones of the rostrum, have been interpreted as bite wounds incurred during dog-on-dog fighting [25]. It is unknown, however, how the frequency of such wounds compare to that observed in northern wolves, and how such patterns might be informative about human-dog relationships.

Antemortem tooth loss and fracture patterns may provide insights on the earliest processes of domestication, dogs' food acquisition processes, and even intentional tooth removal. Some scholars have argued that prior to the first intentional human steps towards domestication, a subset of wolves began to regularly scavenge on human kills and other waste, bringing them into close association with people, which in effect preselected these animals for domestication [26]. Tooth fracture and loss during the life of a canid in such settings should be greater than that seen in hunting wolves, as the former would have to masticate much more bone to extract necessary nutrients because of having secondary access to carcasses [15]–[17].

Tooth loss and fracture frequency in canids might also vary between sexes, and with human husbandry practices. Some studies have indicated that male dogs and wolves tend to out-perform their female counterparts in certain forms of hunting [27]–[29], and that male wolves appear to be more likely than females to experience traumatic injuries [30]. Research on North American gray wolves also indicated significant differences in tooth size between males and females, and suggested this might relate to different degrees of carcass processing between the sexes [31]. Tooth loss and fracture in dogs also could be affected by human provisioning practices, including the degree to which dogs were fed frozen meat or bony scraps as opposed to softer tissues. Further, historic accounts from Greenland report that the carnassials of dogs were removed to prevent them from chewing their bindings [20]–[21]. Such practices would leave very visible signs in archaeological specimens.

Enamel hypoplasia is a condition in which defects in the enamel of teeth are produced by disturbances to the process of enamel formation [32]. Enamel hypoplasia has been little studied in archaeological dog remains, despite the many descriptions of such lesions on dog teeth present in veterinary literature [13], [33]–[36]; comparative data on the occurrence of such features in the teeth of wild canids remain rare. Enamel hypoplasia in canid teeth is caused by diseases such as canine distemper, but also by trauma and dietary deficiencies [33], [35], [37]. Such lesions are potentially informative about food stress, injuries to the facial region, and disease history for dogs and wolves in the first months of life when tooth crowns are forming.

This paper presents comparative data necessary for evaluating the occurrence rates and patterning in cranial trauma and tooth loss, fracture, and enamel hypoplasia in archaeological dog remains. To begin, data are provided for 400 wild wolves from boreal and arctic regions of Canada and Russia. Patterns in this wolf data are compared with those for 144 historic dogs from these same northern regions. We then discuss the meanings of the differences found between wolves and dogs, including how such patterns are informative about dog food stress and provisioning practices.

## Materials

**Figure 1:**
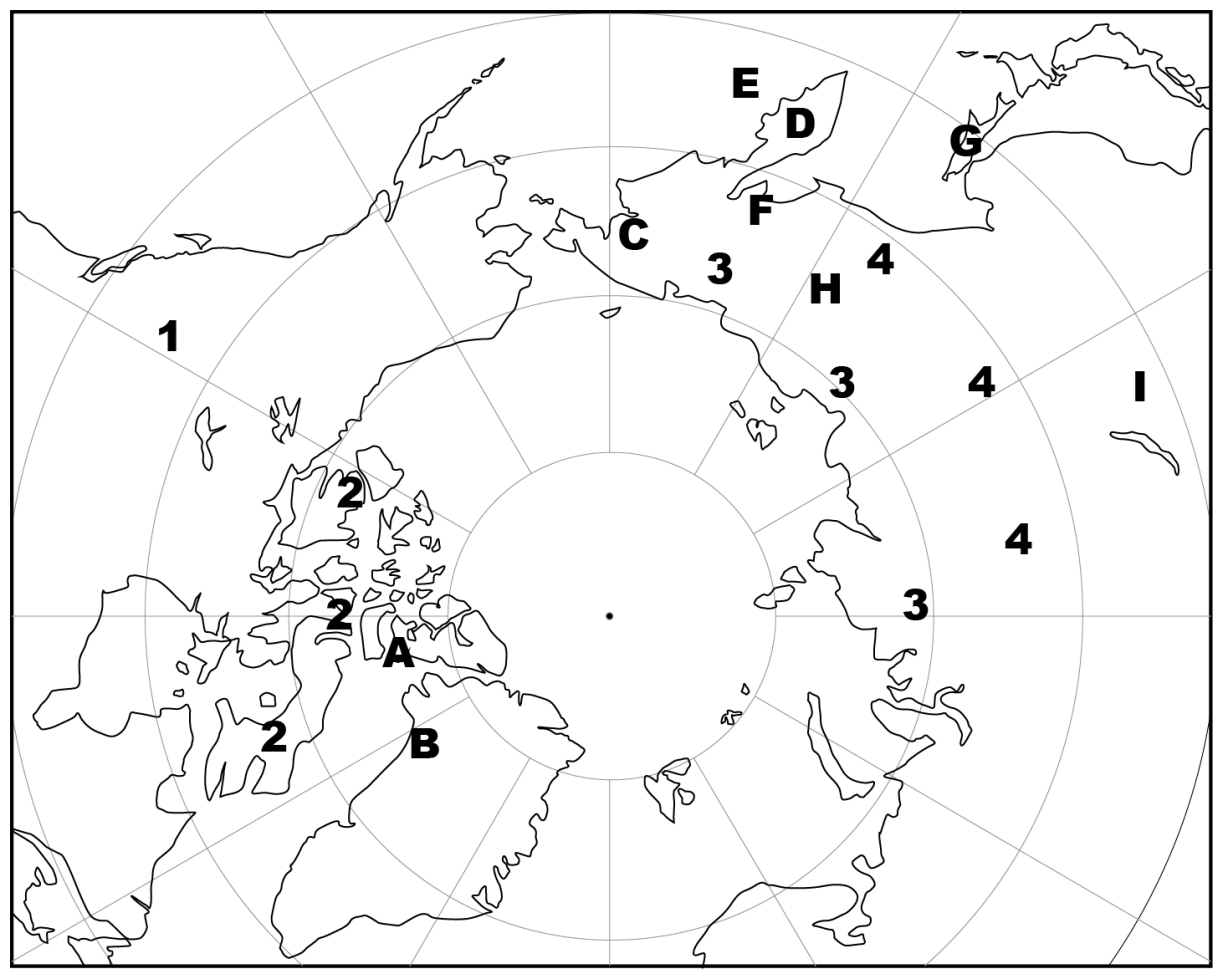
Location of the dog and wolf samples described in this study. Wolves: 1. Alberta, 2. Nunavut, 3. Russian Arctic, 4. Russian Subarctic. Dogs: A. Ellesmere, B. Greenland, C. Chukotka, D. Kamchatka, E. Bering Island, F. Northern Far East, G. Sakhalin, H. Sakha, I. Trans-Baikal.

Summary demographic data for all canid samples analyzed is presented in Table 1, and catalog numbers are listed in Table S1. Wolf crania and mandibles were examined from three collections. The first is from Alberta (Figure 1), consisting of 177 individuals; all are curated at the Royal Alberta Museum (Edmonton, Canada). Nearly all were obtained through poisoning in the 1960s–80s in northern Alberta, which is largely boreal forest, or in the Rocky Mountains [38]. The second collection of wolves, termed the Nunavut group, was assembled from the 1920s–1980s from Arctic Canada (Figure 1) and consists of 131 specimens; all are curated at the Canadian Museum of Nature (CMN; Ottawa, Canada). The final wolf collection, dating from 1885 to 1984, is from Russia, and is subdivided into Arctic (generally north of 66 degrees north latitude, n = 50) and Subarctic (50 to 65 degrees north latitude, n = 42) groups. All the Russian wolves are curated at the Zoological Institute of the Russian Academy of Science (ZIRAS; St. Petersburg, Russia).

**Table 1:** Profile of the specimens analyzed in this study: a. wolves, b. dogs.

Table 1a.					
	Alberta	Nunavut	Russian Subarctic	Russian Arctic	Wolf Totals
	(n = 177)	(n = 131)	(n = 42)	(n = 50)	(n = 400)
**Males**	75	69	20	16	180
**Females**	89	48	15	8	160
**Unknown**	13	14	7	26	60
**Adult**	174	98	42	42	356
**Juvenile**	3	33	0	8	44

Table 1b.					
	Ellesmere	Greenland	Chukotka	Bering Is.	Sakha
	(n = 24)	(n = 13)	(n = 42)	(n = 11)	(n = 21)
**Males**	13	9	26		5
**Females**	11	3	8		1
**Unknown**	0	1	8	11	15
**Adult**	17	13	39	11	19
**Juvenile**	7	0	3	0	2

	Kamchatka	N. Far East	Sakhalin	Trans-Baikal	Dog Totals
	n = 11)	(n = 7)	(n = 9)	(n = 6)	(n = 144)
**Males**	0	6		2	61
**Females**	0	1		1	25
**Unknown**	11	0	7	3	56
**Adult**	6	6	7	6	124
**Juvenile**	5	1	2	0	20

There are nine dog samples from across much of the northern hemisphere (Figure 1). The first set of dog samples includes 24 Inuit sled dogs collected in Grise Fiord, Ellesmere Island (Canada) in 1966–70; most have documented age, sex, and body mass at death and all are curated at the CMN. Dr. M. Freeman, who collected these specimens, was interviewed in 2013. The second dog collection is from northwest Greenland and consists of 13 specimens collected by Robert E. Peary in 1896–7; all are curated at the American Museum of Natural History (AMNH; New York, U.S.A.). All are sled dogs obtained from Greenland Inuit, as reported in Peary's [39] expedition account.

The remaining seven samples of dogs come from the Russian North, and all are housed at the ZIRAS, except for 14 specimens from Chukotka, which are curated at the AMNH. The sample from Chukotka includes 42 dogs collected from 1891 to 1938. The second set of dogs is termed the Bering Island group, collected in 1884. It includes 10 dogs from this island, one of which is listed as a sled dog, and one from nearby Commander Island. The third group includes 11 specimens from unspecified locations on the Kamchatka peninsula in 1884. The fourth group, collected from 1885 to 1928, is from Sakha Republic and includes 21 specimens, with one described as a sled dog, and another as a hare hunting dog. The fifth set of dogs, the Northern Far East group, includes seven specimens collected in 1935. Six are sled dogs, with the seventh being a sled and hunting dog. The sixth group of dogs is from Sakhalin Island, and all were collected from 1882 to 1932. Within this group of nine dogs are six listed as sled dogs. Finally, the seventh group of dogs is from Eastern Trans-Baikal, and consists of six specimens collected in 1914–15.

## Methods

Specimens were selected for study only when the permanent dentition was at least partially erupted and the crowns clearly visible. Those classified as adults had no deciduous dentition in place and the permanent teeth were fully erupted, while those classified as juveniles had either partially unerupted permanent teeth or retained some deciduous teeth. Both dogs and wolves obtain their full adult dentition by approximately six months of age, before adulthood, which occurs in both at roughly two years. However, there presently exists no reliable non-destructive means for assessing the age of the crania and mandibles of dogs or wolves. Ideally, assignment of canids as adults versus juveniles would be done using post-cranial skeletal fusion patterns, but in our dataset all but ∼50 specimens examined lacked postcranial remains. Our ageing approach is a conservative one, and probably under-represents the number of juveniles present in the collections, but it allows us to treat the specimens in all samples consistently. Notably, 129 of the Alberta wolves have age estimates based on counting of cementum bands in the mandibular 1st premolar, and 18 of the Ellesmere dogs have known ages at death (Table S2). All canid crania and mandibles were scored for traits or conditions using standardized data recording forms (see Figure S1). Where possible, patterns were analyzed by age and sex. The Pearson chi-square statistic is used to evaluate differences between or within sample groups. The four primary conditions recorded were:

1. Antemortem tooth loss (AMTL). Teeth were scored as lost antemortem if no evidence of alveolar remodeling could be observed. If root fragments were present but the crown was missing, the tooth was recorded as absent, as it could no longer function for mastication. Alveoli that were completely filled with new bone were counted as lost teeth. This approach could potentially result in congenitally absent teeth being erroneously counted as lost, a point we return to later. Tooth loss was tabulated by tooth type (incisor, canine, premolar, molar; maxillary or mandibular).
2. Fractured teeth. A tooth was scored as fractured if it was broken and the margins of the break edge showed evidence of wear or the jaw showed signs of related infection; fracture occurrences were tabulated by tooth type.
3. Traumatic lesions. The presence of antemortem fractures (including punctures) was recorded by location on the cranium or mandible using the data recording forms.
4. Enamel hypoplasia. Teeth were scored for presence or absence of hypoplastic lesions by tooth type. We counted the presence of such lesions conservatively, identifying the condition as present only when we were fully confident it was present.

## Results

### Antemortem tooth loss

Several overarching patterns are apparent in the AMTL data. First, tooth loss occurs in a far greater percentage of dogs than wolves, with 53.47% and 17.00% (Χ^2^ = 52.85, p = <0.0001) of the total wolf and dog individuals, respectively, having lost at least one tooth (Table 2). Second, dogs of all demographic groups (male, female, adult, juvenile) were more likely to experience tooth loss than their counterparts among the wolves. Third, the overall percentage of teeth lost is also significantly higher in the dogs than in the wolves (4.83% versus 0.91%, respectively; Χ^2^ = 352.22, p = <0.0001), and is higher in the dogs for each tooth type (Table 3). Fourth, the rank order of the teeth most commonly lost is similar in the dogs and wolves, with the three most commonly lost being the upper and lower premolars and the mandibular molars (Table 3).

**Table 2:** Antemortem tooth loss in dogs and wolves by number and percentage of individuals affected: a. wolves, b. dogs.

Table 2a.						
		Alberta	Nunavut	Russian Subarctic	Russian Arctic	Wolf Totals
		(n = 177)	(n = 131)	(n = 42)	(n = 50)	(n = 400)
		n (%)	n (%)	n (%)	n (%)	n (%)
**Sex**	**Male**	14 (18.67)	15 (21.74)	4 (20.00)	2 (12.50)	35 (19.44)
	**Female**	7 (7.87)	8 (16.67)	5 (33.33)	3 (37.50)	23 (14.37)
	**Unknown**	1 (7.69)	3 (21.43)	1 (14.29)	5 (18.52)	10 16.67)
**Age**	**Juvenile**	0 (0.00)	3 (9.09)		0 (0.00)	3 (6.81)
	**Adult**	22 (12.64)	23 (23.47)	10 (23.81)	10 (23.81)	65 (17.81)
**Total**		**22 (12.43)**	**26 (19.85)**	**10 (23.81)**	**10 (20.00)**	**68 (17.00)**

Table 2b.						
		Ellesmere	Greenland	Chukotka	Bering Is.	Sakha
		(n = 24)	(n = 13)	(n = 42)	(n = 11)	(n = 21)
		n (%)	n (%)	n (%)	n (%)	n (%)
**Sex**	**Male**	9 (69.23)	9 (100.00)	14 (53.85)		0 (0.00)
	**Female**	9 (81.82)	3 (100.00)	4 (50.00)		0 (0.00)
	**Unknown**		1 (100.00)	4 (50.00)	4 (36.36)	3 (20.00)
**Age**	**Juvenile**	5 (71.43)		2 (66.67)		0 (0.00)
	**Adult**	13 (54.17)	13 (100.00)	22 (56.41)	4 (36.36)	3 (15.79)
**Total**		**18 (75.00)**	**13 (100.00)**	**24 (57.14)**	**4 (36.36)**	**3 (14.29)**

		Kamchatka	N. Far East	Sakhalin	Trans-Baikal	Dog Totals
		(n = 11)	(n = 7)	(n = 9)	(n = 6)	(n = 144)
		n (%)	n (%)	n (%)	n (%)	n (%)
**Sex**	**Male**		5 (83.33)		1 (50.00)	38 (62.30)
	**Female**		0 (0.00)		0 (0.00)	16 (64.00)
	**Unknown**	5 (45.45)		3 (33.33)	1 (33.33)	21 (37.50)
**Age**	**Juvenile**	0 (0.00)	1 (100.00)	1 (50.00)		9 (45.00)
	**Adult**	5 (83.33)	4 (66.67)	2 (28.57)	2 (33.33)	68 (54.84)
**Total**		**5 (45.45)**	**5 (71.43)**	**3 (33.33)**	**2 (33.33)**	**77 (53.47)**

**Table 3:** Antemortem tooth loss in dogs and wolves by number and percentage of specific tooth group affected: a. wolves, b. dogs.

Table 3a.															
	Alberta	Nunavut	Russian Subarctic		Russian Arctic		Wolf Totals
Tooth Class	n_a_	n_o_	%	n_a_	n_o_	%	n_a_	n_o_	%	n_a_	n_o_	%	n_a_	n_o_	%
Max. incisors	10	1061	0.94	6	786	0.76	1	252	0.4	3	300	1	20	2399	0.83
Man. incisors	9	1062	0.85	4	777	0.51	0	252	0	1	300	0.33	14	2391	0.59
Max. canines	2	354	0.56	1	262	0.38	0	84	0	0	100	0	3	800	0.38
Man. canines	0	354	0	1	259	0.39	0	84	0	0	100	0	1	797	0.13
Max. premolars	2	1412	0.14	19	1044	1.82	3	336	0.89	9	400	2.25	33	3192	1.03
Man. premolars	9	1415	0.64	6	1035	0.58	4	336	1.19	18	400	4.5	37	3186	1.16
Max. molars	0	706	0	1	520	0.19	3	168	1.79	1	200	0.5	5	1594	0.31
Man. molars	5	1060	0.47	21	772	2.72	5	252	1.98	8	295	2.71	39	2379	1.64
**Totals**	**37**	**7424**	**0.5**	**59**	**5455**	**1.08**	**16**	**1764**	**0.91**	**40**	**2095**	**1.91**	**152**	**16738**	**0.91**

Table 3b.															
	Ellesmere	Greenland	Chukotka	Bering Is.	Sakha
Tooth Class	n_a_	n_o_	%	n_a_	n_o_	%	n_a_	n_o_	%	n_a_	n_o_	%	n_a_	n_o_	%
Max. incisors	4	144	2.78	2	78	2.56	0	252	0	8	66	12.12	1	126	0.79
Man. incisors	1	144	0.69	0	72	0	10	234	4.27	6	60	10	1	114	0.88
Max. canines	1	48	2.08	2	26	7.69	3	84	3.57	0	22	0	0	42	0
Man. canines	2	48	4.17	0	24	0	0	78	0	1	20	5	0	38	0
Max. premolars	21	191	10.99	39	104	37.5	13	336	3.87	5	88	5.68	1	168	0.6
Man. premolars	33	190	17.37	25	96	26.04	14	312	4.49	2	80	2.5	1	152	0.66
Max. molars	1	95	1.05	1	52	1.92	6	168	3.57	0	44	0	0	84	0
Man. molars	2	144	1.39	12	72	16.67	12	234	5.13	0	60	0	2	114	1.75
**Totals**	**65**	**1004**	**6.47**	**81**	**524**	**15.46**	**58**	**1698**	**3.42**	**22**	**440**	**5**	**6**	**838**	**0.72**

	Kamchatka	N. Far East	Sakhalin	Trans-Baikal	Dog Totals
Tooth Class	n_a_	n_o_	%	n_a_	n_o_	%	n_a_	n_o_	%	n_a_	n_o_	%	n_a_	n_o_	%
Max. incisors	3	66	4.55	2	42	4.76	0	54	0	5	36	13.89	25	864	2.89
Man. incisors	0	66	0	0	40	0	0	48	0	2	30	6.67	20	808	2.48
Max. canines	0	22	0	0	14	0	0	18	0	1	12	8.33	7	288	2.43
Man. canines	0	22	0	1	14	7.14	0	16	0	1	10	10	5	270	1.85
Max. premolars	3	88	3.41	2	56	3.57	3	72	4.17	2	48	4.17	89	1151	7.73
Man. premolars	5	88	5.68	2	56	3.57	3	64	4.69	2	40	5	86	1078	8.07
Max. molars	0	44	0	0	28	0	1	36	2.78	0	24	0	9	575	1.57
Man. molars	4	66	6.06	6	42	14.29	1	48	2.08	1	30	3.33	40	810	4.94
**Totals**	**15**	**462**	**3.25**	**13**	**292**	**4.45**	**8**	**356**	**2.25**	**14**	**230**	**6.09**	**282**	**5844**	**4.83**

Congenital absence of teeth is unlikely to have a significant impact on our AMTL counts. Antemortem tooth loss can generally be distinguished from dental agenesis or delayed tooth eruption by the presence of alveolar remodeling, which would occur only when teeth are lost during life. When analyzing the canids, congenital absence was identified by both the lack of alveoli and alveolar remodeling. Bilateral absence was also considered as a strong indicator of agenesis. For both sets of canids, agenesis affected only the upper and lower 1^st^ premolars and lower 3^rd^ molars. First premolars previously were reported as occasionally congenitally absent in ‘Eskimo dogs’ [13] and these and lower 3^rd^ molars in some wolves, albeit in low frequencies [9], [10], [40], [18]. However, only 15 of the 144 dogs we examined were suspected of having congenitally absent teeth, and of these nine also showed clear evidence from AMTL. Further, ten wolves also showed bilateral absence of the 1^st^ premolars or lower 3^rd^ molars, which may indicate congenital absence. Among these wolves, only two specimens had AMTL.

Tooth loss also varied among the wolf groups, ranging from a high of 23.81% in the Russian Subarctic sample to a low of 12.43% in the Alberta wolves (Table 2). The percentages of AMTL in the wolf groups do not correlate with latitude—slightly higher rates of loss were seen in Nunavut than Alberta (X^2^ = 2.6581, p = 0.1030), while the more northerly Russian wolves had very slightly lower rates than those from further south (X^2^ = 0.1523, p = 0.6963), but this difference is likely due to chance. Note that a previous study on wolves from across the former Soviet Union reported an AMTL rate (# of individuals affected) of 12.4% [18]. In both the Nunavut and Alberta wolves, tooth loss was greater among males than females, but the difference was only significant in the Alberta sample (Nunavut wolves, X^2^ = 0.3705, p = 0.5427; Alberta wolves, X^2^ = 3.7085, p = 0.0541). The opposite pattern is seen in the Russian wolves, but sample sizes are small and the patterns observed are likely due to chance (subarctic wolves, X^2^ = 0.5926, p = 0.4414; arctic wolves, X^2^ = 1.0343, p = 0.3091). No significant difference in AMTL by sex was reported for the previously published Soviet Union wolf sample [18].

For the dogs, tooth loss varied widely between groups, ranging from a high of 100% of the Greenland specimens being affected, to low of 14.29% for the Sakha specimens (Table 2); only this latter group has tooth loss within the range seen among the wolves. Males and females in the total dog sample experienced roughly the same likelihood of having lost teeth (X^2^ = 0.0082, p = 0.9278), and a slightly higher percentage of adults were affected than juveniles, but this difference was not statistically significant (X^2^ = 0.1389, p = 0.7094), and varied from group to group. The most commonly lost tooth type also varied widely from group to group (Table 3). Note the very high percentages of premolars lost in the Ellesmere and Greenland dogs, which are several orders of magnitude greater than in the other groups, and far above the total dog average for those tooth groups (Ellesmere versus total dog, X^2^ = 14.8895, p = 0.0001; Greenland versus total dog, X^2^ = 105.4026, p< 0.0001).

### Tooth fracture

The tooth fracture data in many ways parallels the AMTL patterns just described. First, the percentage of individuals experiencing tooth fracture (Table 4) is significantly higher among the dogs than the wolves (37.50% opposed to 27.75%, respectively; Χ^2^ = 2.8812, p = 0.0896). The percentage of wolves with fractured teeth observed here is similar to that previously reported for North American wolves (29%; [15]). Second, all dog demographic categories show higher percentages of tooth fracture than their wolf counterparts. Third, dogs and wolves show the same patterns in which teeth are most commonly fractured; the canines and upper incisors being most commonly affected (Table 5).

**Table 4:** Antemortem tooth fracture in dogs and wolves by number and percentage of individuals affected: a. wolves, b. dogs.

Table 4a.						
		Alberta	Nunavut	Russia Subarctic	Russian Arctic	Wolf Totals
		(n = 177)	(n = 131)	(n = 42)	(n = 50)	(n = 400)
		n (%)	n (%)	n (%)	n (%)	n (%)
**Sex**	**Male**	30 (40.00)	18 (26.09)	4 (20.00)	5 (31.25)	57 (31.67)
	**Female**	22 (24.72)	9 (18.75)	1 (6.67)	4 (50.00)	36 (22.50)
	**Unknown**	1 (7.69)	2 (14.29)	2 (28.57)	3 (11.11)	8 (13.33)
**Age**	**Juvenile**	0 (0.00)	0 (0.00)		1 (12.50)	1 (2.27)
	**Adult**	53 (30.46)	29 (29.59)	7 (16.67)	11 (26.19)	100 (28.09)
**Total**		**53 (29.94)**	**29 (22.14)**	**7 (16.67)**	**12 (24.00)**	**111 (27.75)**

Table 4b.						
		Ellesmere	Greenland	Chukotka	Bering Is.	Sakha
		(n = 24)	(n = 13)	(n = 42)	(n = 11)	(n = 11)
		n (%)	n (%)	n (%)	n (%)	n (%)
**Sex**	**Male**	9 (69.23)	8 (88.89)	11 (42.31)		0 (0.00)
	**Female**	4 (36.36)	3 (100.00)	1 (12.50)		0 (0.00)
	**Unknown**		0 (0.00)	1 (12.50)	4 (36.36)	4 (26.67)
**Age**	**Juvenile**	0 (0.00)		1 (33.33)		0 (0.00)
	**Adult**	13 (76.47)	11 (84.62)	12 (30.77)	4 (36.36)	4 (21.05)
**Total**		**13 (54.17)**	**11 (84.62)**	**13 (30.95)**	**4 (36.36)**	**4 (19.05)**

		Kamchatka	N. Far East	Sakhalin	Trans-Baikal	Dog Totals
		(n = 11)	(n = 7)	(n = 9)	(n = 6)	(n = 144)
		n (%)	n (%)	n (%)	n (%)	n (%)
**Sex**	**Male**		1 (16.67)		1 (50.00)	30 (49.18)
	**Female**		0 (0.00)		0 (0.00)	8 (32.00)
	**Unknown**	4 (36.36)		3 (33.33)	0 (0.00)	16 (28.57)
**Age**	**Juvenile**	0 (0.00)	0 (0.00	0 (0.00		1 (5.00)
	**Adult**	4 (66.67)	1 (16.67)	3 (42.86)	1 (16.67)	53 (42.74)
**Total**		4 (36.36)	1 (16.67)	3 (33.33)	1 (16.67)	54 (37.50)

**Table 5:** Antemortem tooth fracture in dogs and wolves by number and percentage of specific tooth group affected: a. wolves, b. dogs.

Table 5a.															
	Alberta	Nunavut	Russian Subarctic		Russian Arctic	Wolf Totals
Tooth Class	n_a_	n_o_	%	n_a_	n_o_	%	n_a_	n_o_	%	n_a_	n_o_	%	n_a_	n_o_	%
Max. incisors	32	1019	3.14	23	732	3.14	4	232	1.72	6	277	2.17	65	2260	2.88
Man. incisors	18	1017	1.77	10	718	1.39	0	236	0.00	1	279	0.36	29	2250	1.29
Max. canines	18	323	5.57	10	237	4.22	2	58	3.45	4	91	4.40	34	709	4.80
Man. canines	11	342	3.22	14	247	5.67	2	84	2.38	2	98	2.04	29	771	3.76
Max. premolars	20	1361	1.47	12	1003	1.20	4	315	1.27	2	376	0.53	38	3055	1.24
Man. premolars	8	1138	0.70	9	991	0.91	1	320	0.31	2	375	0.53	20	2824	0.71
Max. molars	5	703	0.71	6	515	1.17	0	164	0.00	5	198	2.53	16	1580	1.01
Man. molars	12	995	1.21	2	719	0.28	1	230	0.43	2	271	0.74	17	2215	0.77
Totals	124	6898	1.80	86	5162	1.67	14	1639	0.85	24	1965	1.22	248	15664	1.58

Table 5b.															
	Ellesmere	Greenland	Chukotka	Bering Is.	Sakha
Tooth Class	n_a_	n_o_	%	n_a_	n_o_	%	n_a_	n_o_	%	n_a_	n_o_	%	n_a_	n_o_	%
Max. incisors	5	140	3.57	8	66	12.12	8	198	4.04	1	34	2.94	2	91	2.20
Man. incisors	2	143	1.40	1	61	1.64	4	184	2.17	1	41	2.44	3	87	3.45
Max. canines	8	47	17.02	7	24	29.17	5	65	7.69	5	15	33.33	0	36	0.00
Man. canines	10	46	21.74	5	26	19.23	13	68	19.12	3	15	20.00	0	34	0.00
Max. premolars	7	170	4.12	6	56	10.71	7	267	2.62	1	70	1.43	2	140	1.43
Man. premolars	2	156	1.28	7	65	10.77	3	266	1.13	0	67	0.00	0	142	0.00
Max. molars	1	92	1.09	1	46	2.17	5	148	3.38	2	41	4.88	0	80	0.00
Man. molars	4	142	2.82	16	47	34.04	1	189	0.53	1	49	2.04	0	98	0.00
Totals	39	936	4.17	51	391	13.04	46	1385	3.32	14	332	4.22	7	708	0.99

	Kamchatka	N. Far East	Sakhalin	Trans-Baikal	Dog Totals
Tooth Class	n_a_	n_o_	%	n_a_	n_o_	%	n_a_	n_o_	%	n_a_	n_o_	%	n_a_	n_o_	%
Max. incisors	2	57	3.51	0	36	0.00	1	50	2.00	2	29	6.90	29	701	4.14
Man. incisors	0	60	0.00	0	35	0.00	0	47	0.00	2	26	7.69	13	684	1.90
Max. canines	3	20	15.00	0	13	0.00	1	18	5.56	0	10	0.00	29	248	11.69
Man. canines	1	21	4.76	0	13	0.00	0	14	0.00	0	9	0.00	32	246	13.01
Max. premolars	1	80	1.25	0	54	0.00	0	69	0.00	2	41	4.88	26	947	2.75
Man. premolars	0	82	0.00	0	53	0.00	0	59	0.00	0	37	0.00	12	927	1.29
Max. molars	2	42	4.76	2	28	7.14	0	35	0.00	1	24	4.17	14	536	2.61
Man. molars	1	56	1.79	0	35	0.00	0	43	0.00	0	27	0.00	23	686	3.35
Totals	10	418	2.39	2	267	0.75	2	335	0.60	7	203	3.45	178	4975	3.58

Tooth fracture frequency did not always correlate with rates of AMTL. The lowest level of AMTL was seen in the Alberta wolves, but this group has the highest percentage of individuals with fractured teeth, at 29.94% (compare tables 2 and 4). Conversely, the Russian Subarctic wolves had the highest number of specimens with AMTL but have the lowest percentage of individuals with tooth fracture. Overall, more male wolves suffered tooth fracture than females (X^2^ = 2.6022, p = 0.1067), with only Subarctic Russian wolves having more females than males affected (Table 4) but the sample sizes in the latter case are small and the differences likely due to chance (X^2^ = 0.4, p = 0.5271). In all groups where juveniles were present, they are less likely to have broken teeth than adults.

In contrast to the pattern observed in the wolves, the two groups of dogs showing the highest levels of tooth loss, namely those from Ellesmere and Greenland, also show the highest number of individuals with tooth fracture (compare Tables 2 and 4). Males again are more likely to show tooth fracture than females in the total dog sample (X^2^ = 1.1845, p = 0.2764), the single exception being the Greenland sample, which contains too few females to be meaningful. Substantial variability in the number of individuals with tooth fracture is also present among the dog groups, with three (Sakha, N. Far East, and Trans-Baikal) having percentages within the range of that observed in the wolves. Finally, the Ellesmere, Greenland, Chukotka, Bering Island, Kamchatka, and Sakhalin groups all have canines more commonly fractured than other tooth types; for the remaining samples, incisors or molars are most commonly fractured (Table 5). Overall, canines are by far the most commonly fractured teeth in the dogs.

### Traumatic lesions

Lesions on the crania and mandibles consistent with trauma were grouped into two categories: bite wounds and other fractures (Tables 6 and 7). Traumatic lesions characterized as bite wounds were round to oval shaped punctures less than 1 cm in diameter. These were sometimes partially healed and nearly refilled with new bone. Most often such marks were found on the snout, which we subdivided into palate and outer rostrum, while a small number were observed on the frontals. The other fractures recorded were larger depression and radiating fractures.

**Table 6:** Traumatic lesions in dogs and wolves by number and percentage of individuals affected, and by type of lesion: a. wolves, b. dogs.

Table 6a.					
	Alberta	Nunavut	Russia Subarctic	Russian Arctic	Wolf Totals
	(n = 177)	(n-131)	(n = 42)	(n = 50)	(n = 400)
	n (%)	n (%)	n (%)	n (%)	n (%)
**Male**	21 (28.00)	10 (7.63)	3 (15.00)	3 (18.75)	37 (20.56)
**Female**	21 (23.60)	13 (9.92)	3 (20.00)	0 (0.00)	37 (23.13)
**Unknown**	1 (7.69)	0 (0.00)	1 (14.29)	4 (15.38)	6 (10.00)
**Juvenile**	0 (0.00)	2 (6.06)		1 (12.50)	3 (6.82)
**Adult**	43 (24.71)	21 (21.43)	7 (16.67)	6 (14.29)	77 (21.62)
**Fractures**	25 (14.12)	5 (3.82)	3 (7.14)	1 (2.00)	34 (8.50)
**Bite marks**	18 (10.17)	18 (13.74)	6 (14.29)	6 (12.00)	48 (12.00)
**Totals**	**43 (24.29)**	**23 (17.56)**	**7 (16.67)**	**7 (14.00)**	**80 (20.00)**

Table 6b.					
	Ellesmere	Greenland	Chukotka	Bering Is.	Sakha
	(n = 24)	(n = 13)	(n = 42)	(n = 11)	(n = 21)
	n (%)	n (%)	n (%)	n (%)	n (%)
**Male**	8 (61.54)	6 (46.15)	5 (19.23)		2 (40.00)
**Female**	1 (9.09)	3 (100.00)	3 (37.50)		0 (0.00)
**Unknown**		1 (100.00)	2 (25.00)	10 (90.91)	6 (40.00)
**Juvenile**	0 (0.00)		0 (0.00)		1 (50.00)
**Adult**	9 (52.94)	10 (76.92)	10 (25.64)	10 (90.91)	8 (42.11)
**Fractures**	7 (29.17)	7 (53.85)	9 (21.42)	10 (90.91)	2 (9.52)
**Bite marks**	4 (16.67)	3 (21.43)	1 (2.38)	3 (27.27)	7 (33.33)
**Totals**	**9 (37.50)**	**10 (76.92)**	**10 (23.81)**	**10 (90.91)**	**9 (42.86)**

	Kamchatka	N. Far East	Sakhalin	Trans-Baikal	Dog Totals
	(n = 11)	(n = 7)	(n = 9)	(n = 6)	(n = 144)
	n (%)	n (%)	n (%)	n (%)	n (%)
**Male**		0 (0.00)		1 (50.00)	22 (36.07)
**Female**		0 (0.00)		0 (0.00)	7 (28.00)
**Unknown**	2 (18.18)		1 (11.11)	1 (33.33)	23 (41.07)
**Juvenile**	1 (20.00)	0 (0.00)	1 (50.00)		3 (15.00)
**Adult**	1 (16.67)	0 (0.00)	0 (0.00)	2 (33.33)	50 (40.32)
**Fractures**	1 (9.09)	0 (0.00)	1 (11.11)	0 (0.00)	37 (28.68)
**Bite marks**	1 (9.09)	0 (0.00)	0 (0.00)	2 (33.33)	21 (16.28)
**Totals**	**2 (18.18)**	**0 (0.00)**	**1 (11.11)**	**2 (33.33)**	**53 (36.81)**

**Table 7:** Traumatic lesions in dogs and wolves by position: a. wolves, b. dogs.

Table 7a.										
	Alberta	Nunavut	Russian Subarctic		Russian Arctic	Wolf Totals
	n	%	n	%	n	%	n	%	n	%
**Bite mark location**										
External rostrum	8	4.52	7	5.34	3	7.14	1	2.00	19	4.75
Palate	10	5.65	13	9.92	4	9.52	5	10.00	32	8.00
Frontals	4	2.26	4	3.05	1	2.38	0	0.00	9	2.25
**Other fracture location**										
Rostrum	16	9.04	3	2.29	1	2.38	0	0.00	20	5.00
Frontals	3	1.69	2	1.53	2	4.76	1	2.00	8	2.00
Occipital	5	2.82	0	0.00	0	0.00	0	0.00	5	1.25
Mandible	3	1.69	0	0.00	0	0.00	1	2.00	4	1.00

Table 7b.										
	Ellesmere	Greenland	Chukotka	Bering Is.	Sakha
	n	%	n	%	n	%	n	%	n	%
**Bite mark location**										
External rostrum	2	8.33	1	7.69	0	0.00	0	0.00	1	4.76
Palate	3	12.50	2	15.38	1	2.38	3	27.27	6	28.57
Frontals	0	0.00	0	0.00	0	0.00	0	0.00	0	0.00
**Other fracture location**										
Rostrum	1	4.17	2	15.38	4	9.52	5	45.45	0	0.00
Frontals	6	25.00	6	46.15	6	14.29	7	63.64	1	4.76
Occipital	0	0.00	0	0.00	0	0.00	1	9.09	0	0.00
Mandible	0	0.00	0	0.00	0	0.00	1	9.09	1	4.76

	Kamchatka	N. Far East	Sakhalin	Trans-Baikal	Dog Totals
	n	%	n	%	n	%	n	%	n	%
**Bite mark location**										
External rostrum	1	9.09	0	0.00	0	0.00	0	0.00	5	3.88
Palate	1	9.09	0	0.00	0	0.00	2	33.33	18	13.95
Frontals	0	0.00	0	0.00	0	0.00	0	0.00	0	0.00
**Other fracture location**										
Rostrum	0	0.00	0	0.00	0	0.00	0	0.00	12	9.30
Frontals	1	9.09	0	0.00	1	11.11	0	0.00	28	21.71
Occipital	0	0.00	0	0.00	0	0.00	0	0.00	1	0.78
Mandible	0	0.00	0	0.00	0	0.00	0	0.00	2	1.55

As with the previous two dental trauma indices, dogs were more likely than wolves to have traumatic lesions (36.8% versus 20%, respectively; Χ^2^ = 12.22, p = 0.0005), and this pattern was found across all demographic subdivisions (Table 6). When the trauma is divided into bite wounds and other fractures (Table 6), the number of individuals with bite marks differs by only 4.28% between the wolves and dogs (Χ^2^ = 0.5591, p = 0.4546); this difference may be due to chance. The other fractures, however, are found in over three times more dogs than wolves (28.7% versus 8.5%, respectively; Χ^2^ = 23.9992, p = <0.0001). When the traumatic lesions are broken down by affected area (Table 7), both wolves and dogs experienced more bite wounds on their palates than in other places on the skull, but only the wolves showed such lesions on the frontal bones, and this only rarely. The patterning in the other fractures differs strongly between the dogs and wolves. Of the 37 wolves with skull fractures, 20 occurred in the rostrum and 8 in the frontals. Among the 43 dogs with skull fractures, 12 were in the rostrum and 28 were in the frontals. Fractures in other portions of the skull were rare in both groups.

Within the wolves, there is little clear patterning in the occurrences of traumatic lesions, other than those just mentioned. Juveniles are always less likely to have such lesions than adults, but there is no consistency by sex. Bite marks were more common than other fractures in all of the wolf groups, with the exception of the Alberta sample. Further, the occurrence of bite marks was fairly similar in all samples, ranging from 10.2% to 14.3%. Both lower latitude samples (Alberta and Subarctic Russia) had higher incidences of individuals with fractures than in the more northerly samples from their respective continents.

Substantial variability in lesion frequency is present between the groups of dogs analyzed. First, four groups of dog (Chukotka, Kamchatka, N. Far East, and Sakhalin) all have traumatic lesions at or below the level seen in the wolves (Table 6). Within this low trauma group, however, the Chukotka group still has a far higher rate of other fractures (bite marks excluded) than the wolves, with 21.42% of the individuals affected compared to only 8.50% of the wolves displaying such lesions (X^2^ = 6.5304, p = 0.0106). The Ellesmere, Greenland, and Bering Islands dogs all have high percentages of individuals affected by fractures (from 29.2 to 90.9%), which accounts for their overall high percentages of trauma. In this set of dogs with high fracture occurrences, the percentage of individuals with frontal fractures is consistently high, ranging from 25% in the Ellesmere group, to 63.5% of the Bering Island group (Table 7). The two remaining groups, from Sakha and Trans-Baikal, both have high rates of trauma due to the number of individuals affected with bite marks.

### Enamel hypoplasia

**Figure 2:**
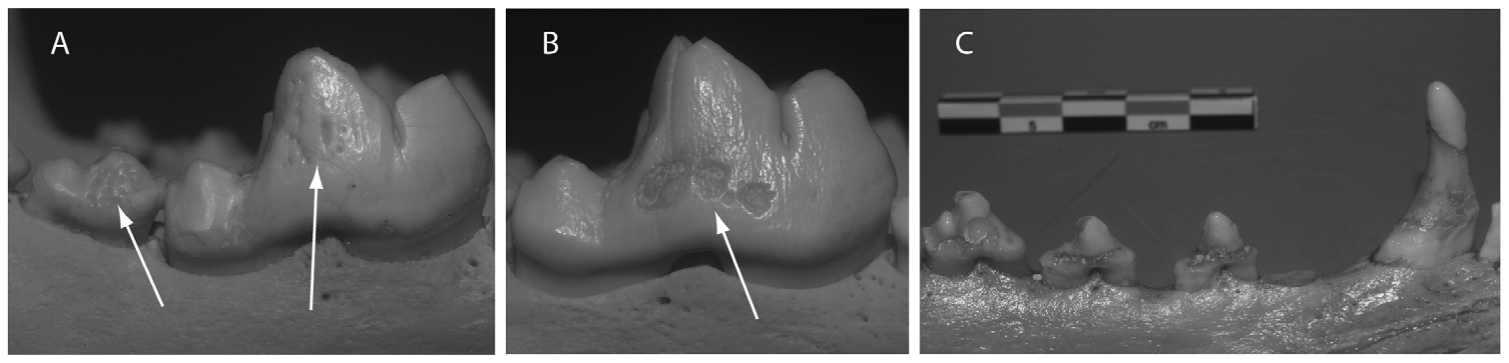
Examples of hypoplastic lesions on wolf and dog teeth. A. Pitted type lesions on the right mandibular 1^st^ and 2^nd^ molars, buccal face. B. Plane type lesion on the right mandibular 1^st^ molar. C. Severe plane type hypoplasitic lesions on the left mandibular canine and 1-3^rd^ premolars.

Enamel hypoplasia can be broadly grouped into three categories: furrow-type defects (linear enamel hypoplasia), pit-type defects, and plane-type defects [41]–[2]. Both pit-type and plane-type defects were observed on the canid teeth (Figure 2) with some affected teeth displaying both types. The defects recorded matched those described in the literature for dogs and wolves [13], [33]–[36]. No cases of furrow-type defects were observed, nor are we aware of such defects being reported in the canid literature. Instances of pit-type hypoplasia consisted of a nonlinear array of pits on the buccal surface of one or more teeth (Figure 2a). Pitting was most common on the mandibular 1^st^ molars and was often asymmetric. Plane-type lesions consisted of two main types: First, isolated hypoplastic lesions exposed broad patches of the underlying enamel (Figure 2b). Like the pit-type defects, these patchy plane-type lesions occurred most frequently on the mandibular carnassials and were generally bilateral. A second, more severe form of plane-type hypoplasia was also observed (Figure 2c). In these cases enamel loss was prolific, affecting multiple teeth with large sections of dentine exposed on all sides of the crowns. All plane-type defects were commonly surrounded by discolored areas of intact enamel.

Overall, occurrences of enamel hypoplasia in the canids were rare, with only 17 dogs and wolves affected in the total sample of 544 individuals (Table 8). A slightly higher percentage of dogs was affected than wolves, but the difference was not significant (4.9% and 2.5%, respectively; Χ^2^ = 1.8889, p = 0.1693). The greatest number of teeth affected in a single individual was 17 in an Alberta wolf, and 25 in a Chukotka dog. In both dogs and wolves, mandibular canines were the most commonly affected teeth, but the numbers of affected teeth is too small for these patterns to be considered meaningful (Table 9). Within the affected wolves, four individuals displayed hypoplastic lesions on 1-3 adjacent teeth; the antimeres were not affected. Three other wolves had such lesions on both the left and right 1^st^ molars (carnassials), while the remaining four wolves displayed lesions on multiple teeth of the upper and lower dentition. Three of the seven dogs showing hypoplastic lesions had only 1–3 adjacent unilateral teeth affected. Two dogs have lesions on both the left and right lower 1^st^ molars, as seen in the wolves. The remaining two specimens include the Chukotka dog with widespread lesions in its upper and lower dentition, and a Sakhalin dog (which lacked mandibles) with its left and right upper 4^th^ premolars (carnassials) and 1^st^ molars affected.

**Table 8:** Hypoplastic lesions in dogs and wolves by number of individuals affected: a. wolves, b. dogs.

Table 8a.						
		Alberta	Nunavut	Russia Subarctic	Russian Arctic	Wolf Totals
		(n = 177)	(n = 131)	(n = 42)	(n = 50)	(n = 400)
		n (%)	n (%)	n (%)	n (%)	n (%)
**Sex**	**Male**	3 (4.00)	1 (1.45)	0 (0.00)	1 (6.25)	4 (2.22)
	**Female**	4 (4.49)	0 (0.00)	0 (0.00)	0 (0.00)	4 (2.50)
	**Unknown**	1 (7.69)	0 (0.00)	0 (0.00)	0 (0.00)	1 (1.67)
**Age Cat**	**Juvenile**	0 (0.00)	0 (0.00	0 (0.00)	1 (12.50)	1 (2.27)
	**Adult**	8 (4.60)	1 (1.02)	0 (0.00)	0 (0.00)	9 (2.53)
**Total**		**8 (4.52)**	1 (0.76)	0 (0.00)	1 (2.00)	10 (2.50)

Table 8b.						
		Ellesmere	Greenland	Chukotka	Bering Is.	Sakha
		(n = 24)	(n = 13)	(n = 42)	(n = 11)	(n = 21)
		n (%)	n (%)	n (%)	n (%)	n (%)
**Sex**	**Male**	0 (0.00)	0 (0.00)	2 (7.69)		0 (0.00)
	**Female**	2 (18.18)	0 (0.00)	1 (12.5)		0 (0.00)
	**Unknown**	0 (0.00)	0 (0.00)	0 (0.00)	0 (0.00)	0 (0.00)
**Age Cat**	**Juvenile**	0 (0.00)		1 (33.33)		0 (0.00)
	**Adult**	2 (11.76)	0 (0.00)	2 (5.13)	0 (0.00)	0 (0.00)
**Total**		2 (8.33)	0 (0.00)	3 (7.14)	0 (0.00)	0 (0.00)

		Kamchatka	N. Far East	Sakhalin	Trans-Baikal	Dog Totals
		(n = 11)	(n = 7)	(n = 9)	(n = 6)	(n = 144)
		n (%)	n (%)	n (%)	n (%)	n (%)
**Sex**	**Male**		0 (0.00)		0 (0.00)	2 (3.28)
	**Female**		0 (0.00)		0 (0.00)	3 (12.00)
	**Unknown**	1 (9.09)		1 (11.11)	0 (0.00)	2 (3.57)
**Age Cat**	**Juvenile**	1 (20.00)	0 (0.00)	0 (0.00)	0 (0.00)	2 (10.00)
	**Adult**	0 (0.00)	0 (0.00)	1 (14.29)	0 (0.00)	5 (4.03)
**Total**		**1 (9.09)**	**0 (0.00)**	**1 (11.11)**	**0 (0.00)**	**7 (4.86)**

**Table 9:** Hypoplastic lesions in dogs and wolves by specific tooth group affected: a. wolves, b. dogs.

Table 9a.															
	Alberta	Nunavut	Russian Subarctic		Russian Arctic	Wolf Totals
Tooth Class	n_a_	n_o_	%	n_a_	n_o_	%	n_a_	n_o_	%	n_a_	n_o_	%	n_a_	n_o_	%
Max. incisors	2	1013	0.20	0	732	0.00	0	232	0.00	2	277	0.72	4	2254	0.18
Man. incisors	0	1005	0.00	0	718	0.00	0	236	0.00	0	279	0.00	0	2238	0.00
Max. canines	1	318	0.31	0	237	0.00	0	58	0.00	1	91	1.10	2	704	0.28
Man. canines	4	335	1.19	0	247	0.00	0	84	0.00	0	98	0.00	4	764	0.52
Max. premolars	9	1354	0.66	0	1003	0.00	0	315	0.00	0	376	0.00	9	3048	0.30
Man. premolars	9	1132	0.80	0	991	0.00	0	320	0.00	0	375	0.00	9	2818	0.32
Max. molars	0	703	0.00	0	515	0.00	0	164	0.00	0	198	0.00	0	1580	0.00
Man. molars	15	995	1.51	2	719	0.28	0	230	0.00	0	271	0.00	17	2215	0.77

Table 9b.															
	Ellesmere	Greenland	Chukotka	Bering Is.	Sakha
Tooth Class	n_a_	n_o_	%	n_a_	n_o_	%	n_a_	n_o_	%	n_a_	n_o_	%	n_a_	n_o_	%
Max. incisors	0	140	0.00	0	66	0.00	2	198	1.01	0	34	0.00	0	91	0.00
Man. incisors	0	143	0.00	0	61	0.00	3	184	1.63	0	41	0.00	0	87	0.00
Max. canines	0	47	0.00	0	24	0.00	2	65	3.08	0	15	0.00	0	36	0.00
Man. canines	1	46	2.17	0	26	0.00	3	68	4.41	0	15	0.00	0	34	0.00
Max. premolars	0	170	0.00	0	56	0.00	6	267	2.25	0	70	0.00	0	140	0.00
Man. premolars	0	156	0.00	0	65	0.00	6	266	2.26	0	67	0.00	0	142	0.00
Max. molars	0	92	0.00	0	46	0.00	1	148	0.68	0	41	0.00	0	80	0.00
Man. molars	2	142	1.41	0	47	0.00	8	189	4.23	0	49	0.00	0	98	0.00

	Kamchatka	N. Far East	Sakhalin	Trans-Baikal	Dog Totals
Tooth Class	n_a_	n_o_	%	n_a_	n_o_	%	n_a_	n_o_	%	n_a_	n_o_	%	n_a_	n_o_	%
Max. incisors	0	57	0.00	0	36	0.00	0	50	0.00	0	29	0.00	2	701	0.29
Man. incisors	3	60	5.00	0	35	0.00	0	47	0.00	0	26	0.00	6	684	0.88
Max. canines	0	20	0.00	0	13	0.00	0	18	0.00	0	10	0.00	2	248	0.81
Man. canines	0	21	0.00	0	13	0.00	0	14	0.00	0	9	0.00	4	246	1.63
Max. premolars	0	80	0.00	0	54	0.00	2	69	2.90	0	41	0.00	8	947	0.84
Man. premolars	0	82	0.00	0	53	0.00	0	59	0.00	0	37	0.00	6	927	0.65
Max. molars	0	42	0.00	0	28	0.00	2	35	5.71	0	24	0.00	3	536	0.56
Man. molars	0	56	0.00	0	35	0.00	0	43	0.00	0	27	0.00	10	686	1.46

## Discussion

Overall, the dogs examined in this study experienced significantly higher rates of tooth loss, tooth fracture, and traumatic lesions than did wolves living in the same ecological regions. They also exhibited slightly higher frequencies of enamel hypoplasia. These patterns could be tied to a number of canid behaviors and life history parameters, but also human husbandry practices.

As the likelihood of experiencing injury increases with age, it is important to rule out age differences as an explanation for the contrasts observed between wolves and dogs. We do not believe that age is an important factor in our study for two reasons. First, the juvenile dogs examined showed rates of tooth loss far higher than those of the juvenile wolves (Table 2); numbers related to tooth fracture are too small to be meaningful, but the percentage affected also is higher in the dogs than the wolves (Table 4). More specific age at death information is available for most of the Alberta wolves and Ellesmere dogs (Table S2). Using this demographic data, half of the Ellesmere dogs can be classified juveniles (two years of age or less), and five of these nine juveniles (55.56%) experienced tooth loss prior to death (excluding cases where congenital absence or non-eruption was suspected), and two of the nine (22.22%) also suffered tooth fractures. The juvenile Alberta wolves showed far fewer individuals affected by tooth loss and fracture (both at 4.34%; Table S2). Second, other modern working dogs seem to have life spans much like those of wolves. For example, sled dogs in Antarctica sharply decline in their working abilities after about 6–7 years of age, if not earlier [43]. Such declining dogs were typically culled, with only a few above this age kept for breeding purposes. Culling of elderly or “worn-out” dogs was also reported in Greenland [21]. Bogoras [44] states that sled dogs in the Chukotka area decline after six to seven years of age, but some work until ten or eleven years old. Detailed quantitative studies of modern Nicaraguan hunting dogs reported an average age at death of 3.7 years, with only 11% of individuals reaching eight years of age [27]; similarly short lives for dogs are reported in other studies [45]–[48]. Data on average age at death for wild wolves is rare, but it appears that few reach seven to eight years of age [49], [50]. Only three of the 129 Alberta wolves reached eight years of age (Table S2).

Differences in feeding practices of dogs and wolves may account for some of the different levels of tooth fracture and loss observed, and perhaps also some of the patterning in traumatic lesions. Relevant aspects of feeding include a series of inter-related factors such as how food is obtained, prey body size, how completely prey/food is being consumed, and the qualities (density, texture) of what is being masticated. We first consider prey size, which might be related to trauma experiences as well as tooth loss and fracture, with larger prey being more capable of striking the canids with forces sufficient to fracture skulls and teeth, and with larger prey having more robust skeletons that would place higher loads on teeth, both during capture and consumption.

Wolves inhabiting the study regions historically have variable diets [49], [51], making it difficult to make simple comparisons between typical prey sizes by region. In Alberta, wild prey potentially encountered by wolves range in size from elk (*Cervus elaphus*) and moose (*Alces alces*) to caribou (*Rangifer tarandus*) and deer (*Odocoileus* spp.), but small mammals such as hare (*Lepus* spp.) also are taken. Wolves in Alberta also prey upon cattle, horse, and sheep [38], [52]. For the Nunavut wolves, average prey size appears to have been smaller than in Alberta, with the most important prey being caribou, but muskox (*Ovibos moschatus*) are also occasionally taken, as are small mammals [53]. For the Russian Subarctic, diets seem to vary generally by latitude, with wolves inhabiting the northern portions of the boreal forest (taiga) relying primarily on hare (*Lepus* spp.)[49], [54]. In the southerly portions of the boreal zone, elk and moose appear to be the most important prey, but smaller deer, wild boar (*Sus scrofa*), rodents, cattle, sheep, and horse also are preyed upon [51]. In Arctic Russia, reindeer (wild or domestic) are the predominant prey, but moose, fox (*Vulpes* spp.), hare, rodents, and ptarmigan (*Lagopus* spp.) also form part of the diet of some wolves [49].

The dogs analyzed in this study appear to have been fed strikingly different diets than their wild counterparts, including substantial quantities of marine mammals and fish. For example, the dogs of Cape York, Greenland subsisted largely on a diet of meat, blubber, and skin of sea mammals (seals (Phocidae) and walrus (*Odobenus rosmarus*))[20]. The dogs were at times fed frozen chunks of these animals, some with pieces of bone in them, which the dogs would gnaw in order to swallow. When the pieces fed to the dogs were unfrozen, they were swallowed as quickly as possible, without being gnawed. Jensen [21] mentions that some Greenland dogs were fed shark meat in summer. Degerbøl and Freuchen [20] further report that in summer, dogs were fed only once a week, or less frequently. In East Greenland, some dogs were left on islands in summer to fend for themselves [55], a practice also reported for other regions of the North American Arctic [56]–[57]. Peary [39] reports feeding his Greenland sled dogs primarily walrus “meat”, but also mentions occasionally provisioning the dogs with seal, polar bear (*Ursus maritimus*), and even other dogs, the latter dying from exhaustion and exposure during his travels. M. Freeman, who collected the Ellesmere dogs, recalled them being fed almost entirely on sea mammal blubber, meat, and skin [see also 58]. Riewe [59] similarly reports dogs at Grise Fiord subsisting primarily on marine mammals. The Inuit of the central Canadian Arctic in the early historic period are said to have fed their dogs seal parts, including internal organs, skins, and bones in winter [56].

Aquatic foods also dominate the diets of dogs from the study regions of Russia represented in the paper. Bogoras [44], who collected a portion of our Chukotka dog sample, states that the “Kamchadal, Koryak, and Russian dogs are fed exclusively on fish – raw, dried, or frozen, according to the season or the locality.” Chukchi dogs, by contrast, were fed seal intestines and blubber from seals, walrus, and whales, but rarely received meat, which was reserved for their owners [44]. The solely blubber diet was considered insufficient for the dogs, and the preferred diet also occasionally included some dried fish or marine mammal meat. Bogoras [44] further reports that dogs were not fed in summer, and that during this time they relied totally on killing or scavenging, which in some cases involved catching rodents, and in others feeding on remnants of salmon (*Oncorhychus* spp.). Yukaghir dogs (in Chukotka and Sakha Republic) are described as subsisting predominantly on fish [60]. The Nanai of the Amur River basin fed their dogs mainly fish, but also occasionally parts of land mammals, typically cooked bones or intestines [22].

Overall, ethnographic and historic accounts of dog diets in these regions suggest they were not being intentionally fed substantial quantities of dense bone that might have contributed to the high levels of tooth loss and fracture observed. Some of these dogs clearly were occasionally scavenging, and at some periods were largely self-sufficient and likely under food stress. Scavenging often involves extracting nutrients from remnants of food items such as discarded animal carcasses and individual bones. Extensive mastication of bone and other hard foods to extract nutrients has been shown to correlate with high rates of tooth fracture in carnivores [12], [15]–[17], and has been specifically linked in studies of fossil carnivores to food stress, including high levels of competition between individuals for prey [16]–[17]. Presumably, high fracture rates would in turn lead to high rates of antemortem tooth loss, as fractures allow bacteria to enter the pulp cavity and can lead to infection of the tooth socket and subsequent tooth loss. Such conditions of periodic food stress and high competition between dogs scavenging for meager and hard to access nutrients might well account for the high levels of tooth loss and fracture observed in most of the northern dog specimens examined. Further, there are no indications that the dogs were commonly encountering larger prey than wolves, which might have led to the higher incidences of trauma observed, including fractures to teeth. Additionally, some of the dog groups (Kamchatka, N. Far East, and Sakhalin) have smaller percentages of individuals with traumatic lesions than seen in all of the wolf groups (Table 6). These dogs nonetheless had higher rates of tooth loss than the wolves (Table 2), which indicates that trauma is not the primary causative factor leading to tooth loss and fracture, at least among these groups.

**Figure 3:**
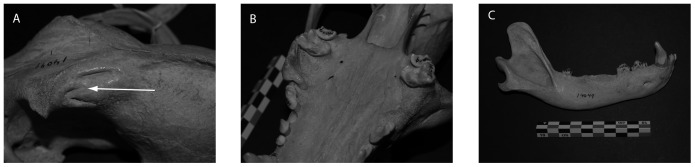
Photographs of a dog crania and mandible collected during the Robert E. Peary expedition in Northwest Greenland in 1897 (specimen #14049, AMNH). A. Healed depression fracture in the left frontal just posterior to the orbit. B. Antemortem loss of the right 4^th^ premolar and antemortem fracture of the left 4^th^ premolar. C. Right mandible with 4^th^ premolar, first molar (carnassial), and 3^rd^ molar lost antemortem. The antemortem tooth loss and fracture observed in this specimen is consistent with intentional tooth removal to inhibit gnawing.

An additional factor contributing to tooth loss among the dogs from Greenland, and perhaps also Ellesmere Island, is intentional tooth removal. Dogs in these regions are reported to have sometimes chewed and ingested their traces and other items made of hide, causing damage to, or loss of, equipment [20]–[21], [61]. To prevent this, dogs in Greenland were sometimes asphyxiated until unconscious and then their teeth crowns filed away or broken off with a stone or metal hammer. In the specimens examined here, this manifests as the bilateral absence or fracture of the upper 3^rd^ and 4^th^ premolars and the lower 1^st^ molars. In some cases, the sheared-off roots or crown remnants are still in place, and the resulting gaps in the dentition are commonly flanked by unmodified teeth (Figure 3b and 3c). Alveoli of such teeth were commonly filled with remodeled bone, but areas of reactive bone and abscessing also were common. In the Greenland dog sample, twelve of the thirteen dogs were suspected of having intentionally removed teeth, while one of the Ellesmere dogs may be affected. The antiquity of this practice should be relatively easy to assess with archaeological dog remains. We are not aware of tooth removal taking place in the Russian North, nor did we see any evidence for them on the specimens from this region; cutting of the tongue has been reported as a preventative measure for such gnawing in parts of the Russian Arctic [60], which would leave no observable trace on the skull.

Our analyses showed that some groups of dogs (from Greenland, Bering Island, Sakha, and Trans-Baikal) have far greater frequencies of bite wounds than the wolves, while others (Chukotka, Kamchatka, N. Far East, and Sakhalin) have far lower frequencies than the wild canids (Table 6). Such bite wounds are likely from other canids, probably other dogs. High numbers of affected individuals could result from fierce competition over food, which we argue was a likely factor in the tooth fracture and loss patterns observed. Further, a high incidence of bite wounds could occur if there were interactions among large numbers of free-ranging dogs, either in human settlements, or where dogs were left unattended on islands. Unfortunately, there is little historical evidence for such practices and behaviors for most of our samples. The Ellesmere dogs were tethered with chains when not pulling sleds, a practice required by law in the 1960s. The percentage of Ellesmere dogs with bite wounds however is similar to that seen in wolves living in this same general region (Table 6). Bogoras [44] mentions that during travel in winter, sled dogs were tethered overnight, but it is not stated whether they were similarly secured during the day. Further, it is unclear which communities he was speaking about, or precisely where he collected the dogs we sampled. Our Chukotka dog sample had some of the lowest rates of bite wounds in the study (Table 6). Overall, no simple correlations between tethering practices and the frequency of bite wounds can be asserted.

Behavioral tendencies in dogs may also influence the frequency of bite wounds and other forms of trauma. Aggressive dogs might be more likely to have traumatic conflicts with other dogs, and perhaps also with humans, resulting in greater numbers of individuals with traumatic lesions. Dog breeds vary in their stereotypical and actual aggressive tendencies [62]–[63], and for some tasks dogs undertake, aggressiveness can be an asset. Such tasks would include providing protection from other humans and predators, and perhaps also hunting where dogs are used to run down and secure prey. How such tendencies and practices varied across our study region is unknown, but some breeds of northern dogs clearly can exhibit aggressive tendencies [64]. Further, a few dog specimens examined were specifically listed as hunting dogs, and many others likely also were involved in such tasks. Interbreeding with wolves, which is occasionally mentioned in northern ethnographies [20], [22], [65], might also have produced animals with aggressive tendencies.

Our data also suggest that traditions of animal discipline involving severe physical force are likewise a factor in shaping the traumatic patterns observed. Dogs from Ellesmere, Greenland, Bering Island, and Chukotka all have high occurrence rates of fractures in their frontal bones, the vast majority being depression fractures (Table 7). These fractures and their positions on the crania are very similar to those described on archaeological dog remains from the Canadian Arctic [23]–[25] and elsewhere [66]. Such fractures are produced by the dogs being struck by blunt objects, and the bone collapsing into the sinuses of the frontal bones (Figure 3a). While such fractures could be caused by kicks from large prey animals, wolves very rarely displayed such lesions (2% of the total wolf population vs. 21.7% across all our dogs). Further, the dogs from Bering Island could not have suffered such lesions as the result of encounters with large terrestrial prey given that, historically, the largest wild mammals on these islands were foxes [67]. This suggests that the depression fractures were caused by people striking the dogs. The groups of dogs with higher rates of frontal fractures also showed higher rates of rostrum fractures than all other dog groups analyzed, and higher rates than in most of the wolves (Table 7). The etiology of these latter lesions is unknown, but blows to the head from humans could also be a cause. Many of these high trauma dogs were used for pulling sleds (the Greenland and Ellesmere dogs) or were from areas where dog sledding was common (Bering Island and Chukotka)[44], [67]. However, the dog groups with low occurrences of all fractures were also from areas where sledding was common, and some groups include multiple individuals recorded as sled dogs (see Materials). In short, the patterns in fractures cannot at present be attributed to the ways dogs were utilized.

Finally, the lack of linear enamel hypoplasia in the permanent teeth of the dogs and wolves observed should not be surprising. Hypoplastic lesions are caused by disruptions in ameloblastic function during tooth crown formation. These disruptions can have many causes, including genetic abnormalities, fevers, viral infections, malnutrition, and localized trauma to the deciduous tooth, all of which can affect the developing teeth in the dental crypt [13]. For dogs and wolves, the permanent crowns form almost simultaneously and very rapidly, within ∼70 to 120 days, in the first few months of life, beginning with the mandibular 1^st^ molar [68]. By approximately 4 months of age, all of the adult crowns are formed. For a linear lesion to form, the disturbances creating it would have to be very acute and short lived, perhaps a week or less. Stresses that are severe enough to cause hypoplastic lesions, such as disease (canine distemper), dietary deficiencies, and even the infection and inflammation associated with trauma, would all likely be far less acute.

Trauma may best explain the cases where dog and wolf specimens showed hypoplastic lesions on 1–3 teeth adjacent teeth (Figure 2a). Systemic stresses like dietary deficiencies or disease would presumably manifest across multiple bilateral teeth. Where bilateral teeth or the entire dentition are affected, systemic stresses are more likely. As we argued earlier, some level of food stress is evidenced in the dogs, and this too seems a possible cause for hypoplastic lesions observed in some specimens. However, it remains impossible to differentiate such lesions from those produced by diseases such as canine distemper. Canine distemper is highly contagious, typically transmitted by aerolization of respiratory fluids carrying the virus, and can infect dogs without producing noticeable symptoms [69]. It has been shown to pass from dogs to wolves (and other species) [69], [70], and has been documented as causing both hypoplastic lesions and lesions on the metaphyses of young dogs [71]–[3]. This disease was clearly present in northern North American and Russia during the general period in which our specimens were collected [57], [60], [70], but its longer history in these regions is unknown.

## Conclusions

Life for some dogs living in recent northern societies included food stress and violent interactions with humans. This pattern was found in societies where subsistence was primarily based on hunting of marine mammals (Ellesmere Island, Greenland), and where other domesticated animals such as reindeer were common (most of the Russian study areas). Food stress manifests in the dogs as high levels of tooth fracture and loss, likely due to scavenging on hard foods; hypoplastic lesions on the teeth may also sometimes mark food stress in young dogs. Such stresses in these dogs, and in ancient ones, should not be surprising. Working dogs, particularly those used for tasks such as pulling sleds, have very elevated metabolic rates due to high activity levels [74]–[75]. Correspondingly, such dogs require significant caloric intake to maintain their energy balance [76]. Keeping dogs can be costly for humans in terms of time and energy—one has to purchase, hunt, fish, and forage in order to provision them [58], and at times these efforts may be unsuccessful or insufficient. Dogs' abilities to withstand food stress and feed themselves are rarely discussed in accounts of archaeological dogs, but these abilities clearly lessen their costs to humans, and were critical to their long-term use in the north and other regions. These abilities of dogs were also likely important in their initial domestication.

Our data indicate that dogs were far more likely to experience some types of fractures than wolves. Some injuries may have been due to encounters with prey, but many also were caused by people intentionally striking dogs, or incurred in accidents. The human niche poses a series of hazards to dogs that are more rarely part of the experiences of wolves. These include humans' possessions—sleds, whips, weapons, and other domesticated animals—all of which have the potential to cause bodily harm to dogs. Clearly, humans themselves also were (and are) a serious hazard faced by dogs, but this remains little discussed in archaeological literature. In some cases, humans modified dog anatomy in order to control their behavior, by intentionally removing chewing teeth to prevent gnawing. Overall, the human-dog relationships inferred from the data are complex and unromantic, but clearly had profound effects on these domesticated animals.

Consideration of the long-term histories of the patterns observed in this study raises a suite of further questions. For example, was food stress in dogs long-standing in the north, and how might this have changed with the introduction of new forms of subsistence, including the use of domesticated reindeer? It also seems likely that changing transportation requirements associated with periods of human colonization or dispersal also would have affected the lives of dogs, but this has been largely ignored in archaeology. Looking beyond the historic past, it seems fully worth exploring the levels of tooth fracture, loss, and enamel hypoplasia among the wolves first undergoing domestication. Is their evidence of food stress in wolves prior to the first steps towards domestication, indicating it was a factor in pushing dogs towards the human niche? It might also be intriguing to examine how patterns of trauma and tooth loss and fracture vary in dogs within larger-scale societies, and by various types of dogs, including feral animals and pets. Human violence towards dogs and other domesticates will always be a contentious issue, but exploring how it varies through time and space will provide a more nuanced picture of human-animal relations in the past.

## Supporting Information

Figure S1 **Data collection forms used in this study.**
(PDF)

Table S1 **Catalog numbers for specimens analyzed in this study.**
(XLSX)

Table S2 **Age structure of the Alberta wolves and Ellesmere dogs, where known.** Antemortem tooth loss and antemortem tooth fracture by age category also shown.
(DOCX)

## References

[1] Bartelle BG, Vellanoweth RL, Netherton ES, Poister NW, Kendig WE, et al (2010) Trauma and pathology of a buried dog from San Nicolas Island, California, U.S.A. . J Archaeolog Sci 37: 2721–2734.

[2] Binois A, Wardius C, Rio P, Bridault A, Petit C (2013) A dog's life: multiple trauma and potential abuse in a medieval dog from Guimps (Charente, France). Int J Paleopath 3: 39–47. 10.1016/j.ijpp.2013.02.001 29539358

[3] Lawler DF, Rubin DA, Evans RH, Hildebolt CF, Smith KE, et al (2013) Differential diagnosis of an unusual shoulder articular lesion in an ancient domestic dog (Canis lupus familiaris L., 1758). Int J Paleopath 3: 282–287. 10.1016/j.ijpp.2013.06.001 29539565

[4] Losey RJ, Bazaliiskii VI, Garvie-Lok S, Germonpré M, Leonard JA, et al (2011) Canids as persons: Early Neolithic dog and wolf burials, Cis-Baikal, Siberia. J Anthropol Archaeol 30: 174–189.

[5] MacKinnon M (2010) ‘Sick as a dog’: zooarchaeological evidence for pet dog health and welfare in the Roman world. World Archaeol 42 (2): 290–309.

[6] MacKinnon M, Belanger K (2006) In sickness and in health: care for an arthritic Maltese dog from the Roman cemetery of Yasmina, Carthage, Tunisia. In: Snyder, L.M, Moore, E.A, (Eds) Dogs and People in Social, Working, Economic or Symbolic Interaction. Oxford: Oxbow Press. pp. 38–43.

[7] Warren DM (2000) Paleopathology of Archaic dogs from the North American Southeast. In: Crockford, S.J. (Ed.), Dogs Through Time: An Archaeological Perspective. Oxford: Archeaeo press. pp. 105–114.

[8] Zinoviev AV (2010) Study of Medieval dogs from Novgorod, Russia (X-XIV Century). Int J Osteoarchaeol 22: 145–157.

[9] Andersone Z, Ozoliņŝ J (2000) Craniometrical characteristics and dental anomalies in wolves Canis lups from Latvia. Acta Theriol 45 (4): 549–558.

[10] Buchalcyzk T, Dynowski J, Szteyn S (1981) Variations in the number of teeth and asymmetry of the skull in the wolf. Acta Theriol 26 (2): 23–30.

[11] Dolgov VA, Rossolimo OL (1964) Dental abnormalities in Canis lupus Linnaeus, 1758. Acta Theriol 8 (16): 237–244.

[12] Leonard JA, Vila C, Fox-Dobbs K, Koch PL, Wayne RK, et al (2007) Megafaunal extinctions and the disappearance of a specialized wolf ecomorph. Curr Biol 17: 1146–1150. 17583509 10.1016/j.cub.2007.05.072

[13] Miles AEW, Grigson C (1990) Colyer's Variations and Diseases of the Teeth of Animals. Cambridge: Cambridge University Press.

[14] Pavlovic D, Gomercic T, Guzvia G, Kusak J, Huber D (2007) Prevalence of dental pathology in wolves (Canis lupus L.) in Croatia – a case report. Veterinarski Arhiv 77 (3): 291–297.

[15] Van Valkenburgh B (1988) Incidence of tooth breakage among large, predatory mammals. Am Nat 131 (2): 291–302.

[16] Van Valkenburgh B (2009) Costs of carnivory: tooth fracture in Pleistocene and recent carnivorans. Biol J Linn Soc 96: 68–81.

[17] Van Valkenburgh B, Hertel F (1993) Tough times at La Brea: tooth breakage in large carnivores of the Late Pleistocene. Science 261 (5120): 456–459. 10.1126/science.261.5120.456 17770024

[18] Vila C, Urios V, Castroviejo J (1993) Tooth losses and anomalies in the wolf (Canis lupus). Can J Zool 71: 968–971.

[19] Wobeser G (1992) Traumatic, degenerative, and developmental lesions in wolves and coyotes from Saskatchewan. J Wildlife Dis 28 (2): 268–275. 10.7589/0090-3558-28.2.268 1602579

[20] Degerbøl M, Freuchen P (1976) Zoology I: Mammals. Reprint of Report of the Fifth Thule Expedition 1921–24, vol. 2, no. 4–5. New York: AMS Press Inc.

[21] Jensen B (1961) Folkways of Greenland Dog Keeping. Folk 3: 43–66.

[22] Samar AP (2010) Traditsionnoe Sobakovodstvo Nanaitsev. Vladivostok: Dal'nauka.

[23] Morey DF (2010) Dogs: Domestication and the Development of a Social Bond. Cambridge: Cambridge University Press.

[24] Morey DF, Aaris-Sørensen K (2002) Paleoeskimo dogs of the Eastern Arctic. Arctic 55 (1): 44–56.

[25] Park RW (1987) Dog remains from Devon Island, N.W.T.: archaeological and osteological evidence for domestic dog use in Thule Culture. Arctic 40 (3): 184–190.

[26] Coppinger R, Coppinger L (2001) Dogs: A New Understanding of Canine Origin, Behavior, and Evolution. Chicago: University of Chicago Press.

[27] Koster JM, Tankersley KB (2012) Heterogeneity of hunting ability and nutritional status among domestic dogs in lowland Nicaragua. P Natl Acad Sci USA 109: E463–E470. 10.1073/pnas.1112515109 PMC3286926 22232662

[28] MacNulty DR, Smith DW, Mech LD, Eberly LE (2009) Body size and predatory performance in wolves: is bigger better? J Anim Ecol 78: 532–539. 19175444 10.1111/j.1365-2656.2008.01517.x

[29] Mech LD (1999) Alpha status, dominance, and division of labor in wolf packs. Can J Zool 77: 1196–1203.

[30] Nielson CA (1977) Wolf Necropsy Report: Preliminary Pathological Observations. Special Report, Federal Aid in Wildlife Restoration Projects W-17-8 and W-17-19. Juneau: Alaska Department of Fish and Game.

[31] O'Keefe FR, Meachen J, Fet EV, Brannick A (2013) Ecological determinants of clinal morphological variation in the cranium of the North American gray wolf. J Mammal 94 (6): 1223–1236.

[32] Hillson S (2005) Teeth. Second edition. Cambridge: Cambridge University Press.

[33] Crossley DA, Penman S (1995) BSAVA Manual of Small Animal Dentistry. 2nd Edition. Gloucestershier: British Small Animal Veterinary Association.

[34] Maxie MG (2007) Pathology of Domestic Animals. 5^th^ edition. Philadelphia: A. Saunders, Ltd.

[35] Mellanby M (1929) Diet and the Teeth: An Experimental Study. Part I Dental Structure in Dogs. London: His Majesty's Stationary Office.

[36] Verstraete FJM (1999) Self-Assessment Color Review of Veterinary Dentistry. Ames: Iowa State University Press.

[37] Dubielzig RR (1979) The effect of canine distemper on the ameloblastic layer of the developing tooth. Vet Pathol 16: , 268–270. 10.1177/030098587901600216 442456

[38] Bjorge RR, Gunson JR (1985) Evaluation of wolf control to reduce cattle predation in Alberta. J Range Manage 38 (6): 483–487.

[39] Peary RE (1898) Northward Over the “Great Ice”. Volumes 1 and 2. New York: Frederick A. Stokes Company.

[40] Räikkönen J, Vucetich JA, Vucetich LM, Peterson RO, Nelson MP (2013) What the inbred Scandinavian wolf population tells us about the nature of conservation. PLoS One 8 (6): e67218 doi:10.1371/journal.pone.0067218 10.1371/journal.pone.0067218 PMC3689695 23805301

[41] Berten J (1895) Hypoplasie des Schmelzes (Congenitale Schmelzdefecte; Erosionen). Deutsche Monatsschrift für Zahnheilkunde 13, 425–439; 483–498; 533–548; 587–606.

[42] Hillson S (1996) Dental Anthropology. Cambridge: Cambridge University Press.

[43] Bellars ARM (1969) Veterinary studies on the British Antarctic Survey's sledge dogs. I. Diseases and Accidents. Brit Antarct Surv B 21: 1–18.

[44] Bogoras W (1909) The Chukchee. Memoirs of the American Museum of Natural History, Volume 11. New York: Johnson Reprint Company.

[45] Fiorello CV, Noss AJ, Deem S L (2006) Demography, hunting ecology, and pathogen exposure of domestic dogs in the Isoso of Bolivia. Conserv Biol 20: 762–771. 16909569 10.1111/j.1523-1739.2006.00466.x PMC7202241

[46] Ikeya K (1994) Hunting with dogs among the San in the central Kalahari. Afr Stud Mono 15: 119–134.

[47] Lupo KD (2011) A dog is for hunting. In: Albarella, U. (Ed.), Ethnozooarchaeology. Oxbow: Oxbow Press. pp. 4–12.

[48] Koster JM (2008) Hunting with dogs in Nicaragua: An optimal foraging approach. Curr Anthropol 49: 935–944.

[49] Bibikov DI (1985) Volk: Proiskhozhdenie, Sistematika, Morfologiia, Ekologiia. Moscow: Nauka. Pp. 609.

[50] Stephenson RO, Johnson LJ (1973) Wolf Report, 1971–1972. Federal Aid in Wildlife Restoration Progress Report XI, W-17-4 and 5. Juneau: Alaska Department of Fish and Game.

[51] Peterson RO, Ciucci P (2003) The wolf as a carnivore. In Mech, L.D., Botani, L. (Eds.) Wolves: Behaviour, Ecology, and Conservation. Chicago: University of Chicago Press. pp. 104–130.

[52] Morehouse AT, Boyce MS (2011) From venison to beef: seasonal changes in wolf diet composition in a livestock landscape. Front Ecol Enviro 9: 440–445.

[53] Krizan J (2006) Nunavut wolf morphology and diet study. Final Wildlife Report No. 11, Wildlife Research Section. Igloolik: Department of Environment.

[54] Labutin IuV (1972) Geograficheskie osobennosti pitaniia volka i lisitsy. In Zoologicheskie Problemy Sibiri. Novosibirsk: Nauka.pp. 413–415.

[55] Weyer EM (1932) The Eskimos, Their Environment and Folkways. New Haven: Yale University Press.

[56] Boas F (1974) The Central Eskimo. Toronto: Coles Publishing Company Limited.

[57] Bohm J, Blixenhrone-Moller M, Lund E (1989) A serious outbreak of canine distemper among sled dogs in Northern Greenland. Arct Med Res 48: 195–203. 2590318

[58] Freeman MMR (1969-70) Studies in maritime hunting I. Ecologic and technologic restraints on walrus hunting, Southampton Island N.W.T. . Folk 11–12: 155–171.

[59] Riewe RR (1977) The utilization of wildlife in the Jones Sound Region by the Grise Fiord Inuit. In: Bliss, L.C. (Ed.), Truelove Lowland, Devon Island, Canada: A High Arctic Ecosystem. Edmonton: University of Alberta Press. pp. 623–644.

[60] Chikachev AG (2004) Ezdovoe Sobakovodstvo Iakutii. Iakutsk: SO RAN.

[61] Croft A (1937) West Greenland sledge dogs. Polar Rec 2 (13): 68–81.

[62] American Kennel Club (1975) The Complete Dog Book. New York: Howell Book House.

[63] Duffy DL, Hsu Y, Serpell JA (2008) Breed differences in canine aggression. Appl Anim Behav Sci 114: 441–460.

[64] Montcombroux G (1997) The Canadian Inuit Dog: Canada's Heritage. Winnipeg: Whipoorwill Press.

[65] MacRury IK (1991) The Inuit Dog: Its Provenance, Environment and History. Masters thesis, Darwin College, University of Cambridge.

[66] Baker J, Brothwell D (1980) Animal Diseases in Archaeology. London: Academic Press.

[67] Jochelson W (1968) History, Ethnology and Anthropology of the Aleut. New York: Humanities Press.

[68] Arnall L (1960) Some aspects of dental development in the dog—I. calcification of crown and root of the deciduous dentitions. J Small Anim Pract 1: 169–173.

[69] Deem SL, Spelman LH, Yates RA, Montali RJ (2000) Canine distemper in terrestrial carnivores: a review. J Zoo Wildlife Med 31: 441–451. 10.1638/1042-7260(2000)031[0441:CDITCA]2.0.CO;2 11428391

[70] Elton C (1931) Epidemics among sledge dogs in the Canadian Arctic and their relation to disease in the arctic fox. Can J Res 5 (6): 673–692.

[71] Baumgartner W, Boyce RW, Weisbrode SE, Aildinger S, Axthelm MK, et al (1995) Histologic and immunocytochemical characterization of canine distemper–associated metaphyseal bone lesions in young dogs following experimental infection. Vet Pathol 32: 702–709. 8592806 10.1177/030098589503200612

[72] Dubielzig RR, Higgins RJ, Krakowka S (1981) Lesions of the enamel organ of developing dog teeth following experimental inoculation of gnotobiotic puppies with canine distemper virus. Vet Pathol 18: 684–689. 7281465 10.1177/030098588101800513

[73] Malik R, Dowden M, Davis PE, Allan GS, Barrs VR, et al (1995) Concurrent juvenile cellulitus and metaphyseal osteopathy: an atypical canine distemper virus syndrome? Aust Vet Pract 25: 62–67.

[74] Hammond KA, Diamond J (1997) Maximal sustained energy budgets in humans and animals. Nature 386: 457–462. 9087402 10.1038/386457a0

[75] Hinchcliff KW, Reinhart GA, Burr JR, Schreier CJ, Swenson RA (1997) Metabolizable energy intake and sustained energy expenditure of Alaskan sled dogs during heavy exertion in the cold. Am J Vet Res 58: 1457–1462. 9401699

[76] Gerth N, Sum S, Jackson S, Starck JM (2009) Muscle plasticity of Inuit sled dogs in Greenland. J Exp Biol 212: 1131–1139. 19329747 10.1242/jeb.028324

