# Supplementary material for: Craniomandibular Trauma and Tooth Loss in Northern Dogs and Wolves: Implications for the Archaeological Study of Dog Husbandry and Domestication
Source: PLoS One. 2014 Jun 18;9(6):e99746. doi: 10.1371/journal.pone.0099746 (PMC4062439; doi:10.1371/journal.pone.0099746)
Supplement: Figure S1 — Data collection forms used in this study. (PDF) [file pone.0099746.s001.pdf]

CANID—CRANIAL TRAUMA & DENTAL DISEASE RECORDING FORM

Observer: ______________________________ Date: ______________________________

Numeric ID: ______________________________ Provenience: ______________________________

Tooth Loss Categories

A= antemortem

P = postmortem

U = unknown

Indicate bone missing AM with cross-hatching

Indicate bone missing PM with wavy lines

CRANIUM—DORSAL VIEW

L 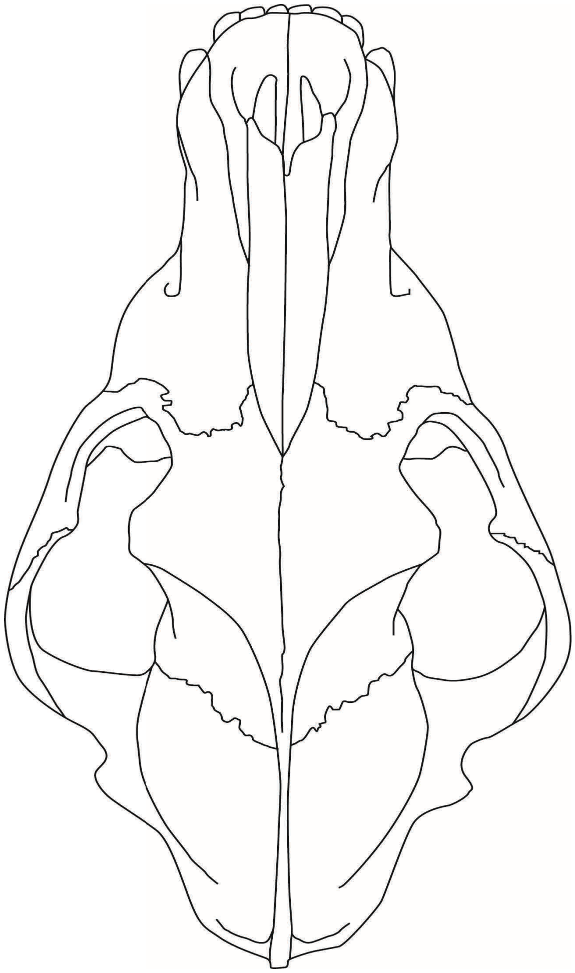 R

CRANIUM—VENTRAL VIEW

R 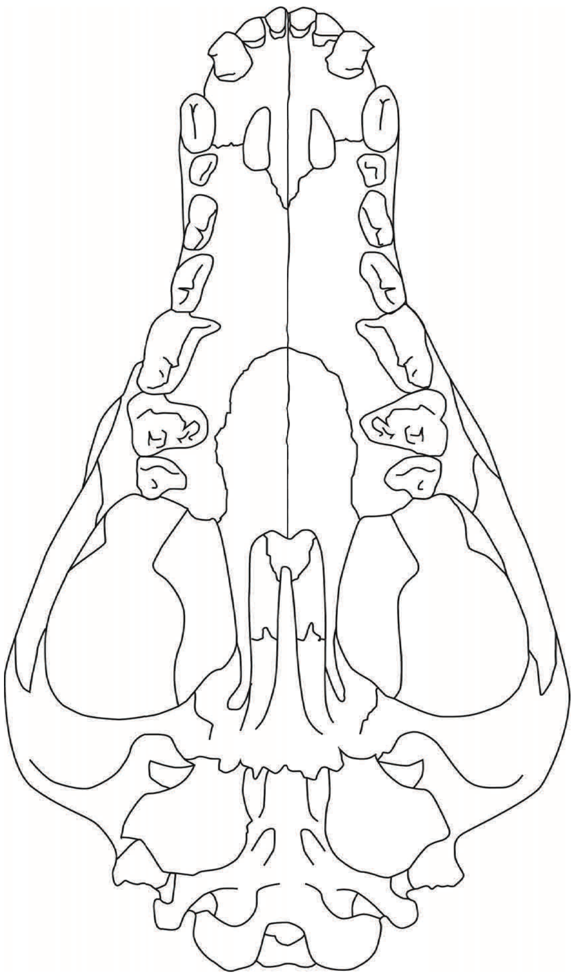 L

MAXILLAE—LINGUAL VIEW

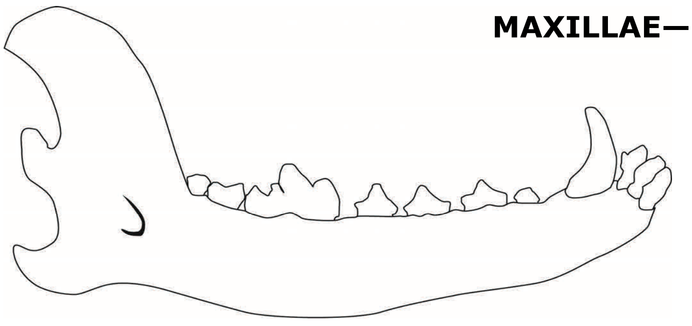

L

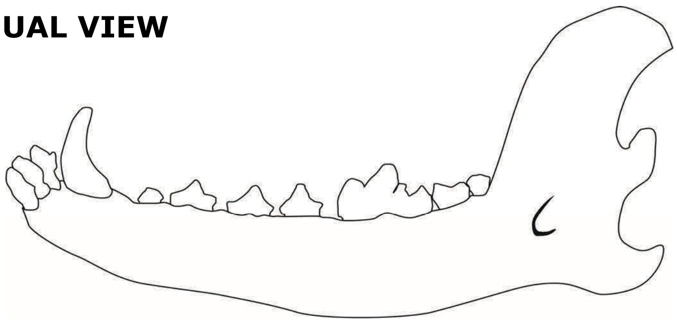

R

CANID—CRANIAL TRAUMA & DENTAL DISEASE RECORDING FORM

Observer: ______________________ Date: ______________________

Numeric ID: ______________________ Provenience: ______________________

Indicate bone missing AM with cross-hatching

Indicate bone missing PM with wavy lines

SKULL—LATERAL/BUCCAL VIEW

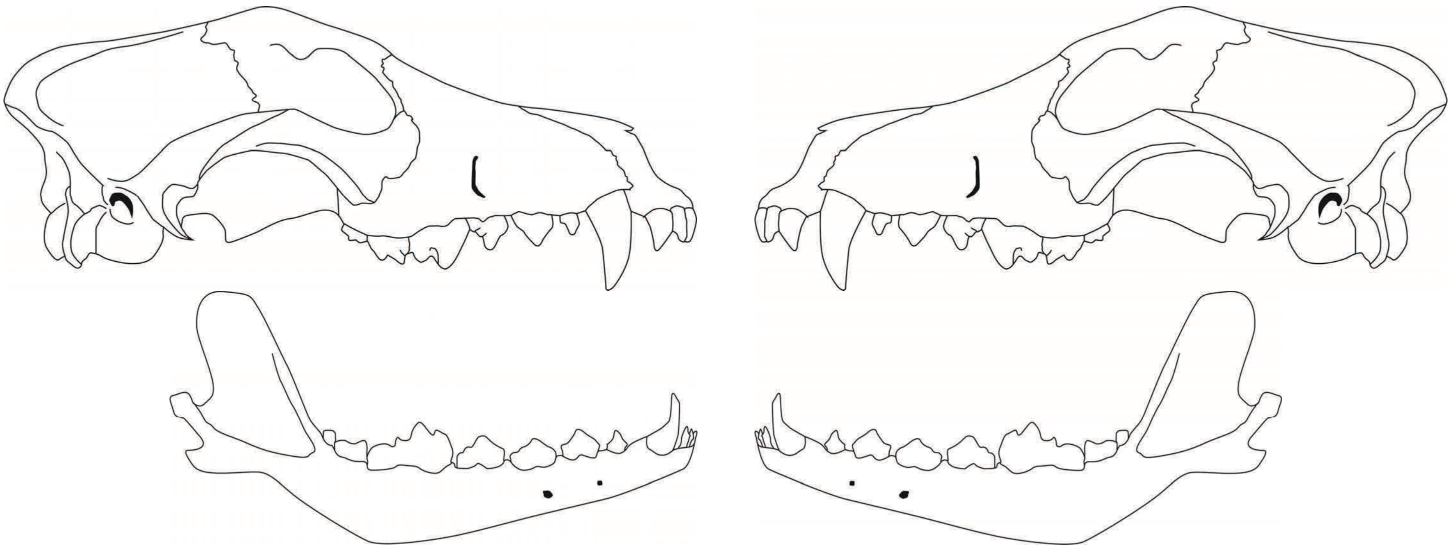

R **L**

Notes: ____

____

____

____

____

____

____

____

**Checklist:**	**AMTL**	**Tooth Fracture**	**Enamel Hypoplasia**	**Periodontal Disease**	**Trauma**	**Osteo-arthritis**	**Poly/Hypo-dontia**
Present	___	___	___	___	___	___	___
Absent	___	___	___	___	___	___	___
Unobservable	___	___	___	___	___	___	___
