# Supplementary material for: Craniomandibular Trauma and Tooth Loss in Northern Dogs and Wolves: Implications for the Archaeological Study of Dog Husbandry and Domestication
Source: PLoS One. 2014 Jun 18;9(6):e99746. doi: 10.1371/journal.pone.0099746 (PMC4062439; doi:10.1371/journal.pone.0099746)
Supplement: Table S2 — Age structure of the Alberta wolves and Ellesmere dogs, where known. Antemortem tooth loss and antemortem tooth fracture by age category also shown. (DOCX) [file pone.0099746.s003.docx]

| **Table S2.** | | | | |
| --- | --- | --- | --- | --- |
| Age structure of Alberta wolves and Ellesmere dogs, where known. | | | | |
| **Alberta wolves** | |  | **Ellesmere dogs** | |
| **Age** | **n** | **%** | **n** | **%** |
| **0.5** | 24 | 18.60 | 5 | 27.78 |
| **1.5** | 22 | 17.05 | 4 | 22.22 |
| **2.5** | 24 | 18.60 | 1 | 5.56 |
| **3.5** | 27 | 20.93 | 1 | 5.56 |
| **4.5** | 13 | 10.08 | 1 | 5.56 |
| **5.5** | 8 | 6.20 | 2 | 11.11 |
| **6.5** | 8 | 6.20 | 2 | 11.11 |
| **7.5** | 2 | 1.55 | 2 | 11.11 |
| **8.5** | 1 | 0.78 | 0 | 0.00 |
| **Total** | 129 |  | 18 |  |
|  |  |  |  |  |
| Antemortem tooth loss by age category, number of individuals affected. | | | | |
| **Alberta wolves** |  |  | **Ellesmere dogs** |  |
| **Age** | **n_a_** | **%** | **n_a_** | **%** |
| **0.5** | 0 | 0.00 | 2 | 40.00 |
| **1.5** | 2 | 9.09 | 3 | 75.00 |
| **2.5** | 4 | 16.67 | 1 | 100.00 |
| **3.5** | 2 | 7.41 | 0 | 0.00 |
| **4.5** | 2 | 15.38 | 1 | 100.00 |
| **5.5** | 2 | 25.00 | 1 | 50.00 |
| **6.5** | 3 | 37.50 | 2 | 100.00 |
| **7.5** | 0 | 0.00 | 1 | 50.00 |
| **8.5** | 0 | 0.00 | 0 | 0.00 |
| **Total** | 15 | 11.63 | 11 | 61.11 |
|  |  |  |  |  |
| Antemortem tooth fracture by age category, number of individuals affected. | | | | |
| **Alberta wolves** |  |  | **Ellesmere dogs** |  |
| **Age** | **n_a_** | **%** | **n_a_** | **%** |
| **0.5** | 1 | 4.17 | 0 | 0.00 |
| **1.5** | 1 | 4.55 | 2 | 50.00 |
| **2.5** | 5 | 20.83 | 1 | 100.00 |
| **3.5** | 12 | 44.44 | 0 | 0.00 |
| **4.5** | 7 | 53.85 | 1 | 100.00 |
| **5.5** | 4 | 50.00 | 0 | 0.00 |
| **6.5** | 4 | 50.00 | 2 | 100.00 |
| **7.5** | 1 | 50.00 | 2 | 100.00 |
| **8.5** | 1 | 100.00 | 0 | 0.00 |
| **Total** | 36 | 27.91 | 8 | 44.44 |
